# AI-Integrated Counseling Administration Quality and Organizational Support as Drivers of Early Risk Detection in Indonesian Schools

**DOI:** 10.12688/f1000research.178853.1

**Published:** 2026-04-19

**Authors:** Novebri Novebri, Dedy Achmad Kurniady, Aan Komariah, Risbon Sianturi, Deni Kadarsah, Badrud Tamam, Riema Afriani Kusumadewi, Dani Darmawan, Akil Akil, Hadiyanto Hadiyanto

**Affiliations:** 1 Universitas Pendidikan Indonesia, Bandung, West Java, 40154, Indonesia; 2 Universitas Wiraloka, Indramayu, 45213, Indonesia; 3 STAI Darussalam Kunir, Subang, 41257, Indonesia; 4 Universitas Singaperbangsa Karawang, Karawang Regency, West Java, 41361, Indonesia; 5 Universitas Negeri Padang, Padang, West Sumatera, 25173, Indonesia

**Keywords:** Artificial Intelligence in Education, School Counseling, Organizational Support, Early Risk Detection, Student Well-being, Quality Education (SDGs 4), Good Health and Well-being (SDGs 3)

## Abstract

Currently, cases of bullying and violence in secondary schools in Indonesia are showing an increasing trend. Therefore, the development of a proactive early detection system focused on the welfare and mental health of students is a primary goal. Current dynamics indicate that the use of AI in education is still dominated by technical, academic, and adaptive learning aspects, while the context of administrative counseling services and early detection of violence in schools has not been fully integrated. This study analyzes the integration of AI, administrative counseling quality, and school support in supporting the early risk detection of students in secondary schools in Indonesia. Using a quantitative approach with a survey design involving 619 students, data were collected through Google Forms and analyzed using SEM-PLS. The results of the research data analysis indicate a positive effect of AI on the quality of administrative counseling, organizational support, and early risk detection. The quality of administrative counseling also has a significant effect on school support and early risk detection, with school support having the strongest effect on early risk detection, with β = 0.497. Furthermore, administrative counseling quality and school support act as mediators of the relationship between AI variables and early risk detection. The novelty of this research lies in the development and testing of an integrative model based on socio-technical systems that places the quality of school counseling and support administration as a key factor in the effectiveness of AI utilization for the prevention of bullying and violence in secondary schools in Indonesia.

## Introduction

Schools, as institutions for learning, should be safe places for students to learn and socialize. However, it’s undeniable that violence remains a frequent issue, both physical and verbal. Behaviours such as bullying, physical fights, and verbal and nonverbal harassment are triggers for increased anxiety, depression, and mental health issues in students ( Arslan et al., 2021; Kaspar, 2013; Kim et al., 2020; Liu et al., 2025; Wang et al., 2024). In addition, this form of violence also causes psycho-social and academic problems for students at school and at home ( Gutzwiller-Helfenfinger, 2021; Kernbach-Wighton, 2022). In recent years, attention to this issue has increased as public awareness of the importance of creating a safe, comfortable, and healthy learning environment for students has grown. However, prevention efforts are often reactive and suboptimal.

Therefore, schools need to manage their student counseling services wisely. Today, technological advances, particularly artificial intelligence, help monitor and analyze student behavior in the classroom, identifying disciplinary violations and bullying early ( Sidhu & Sidhu, 2025; Zheng et al., 2025). Schools have begun adopting technologies such as counseling chatbots and automated notification systems via WhatsApp or SMS. AI can significantly improve mental health services in schools by providing real-time physiological monitoring, emotional regulation, and cognitive behavioral modification ( Guo & Hou, 2021).

AI chatbots can offer immediate, non-judgmental feedback and support, although they should be complemented by human counselors to address issues of empathy and trust ( Al-Shraifin et al., 2025; Chan, 2025). AI should be used to complement and enhance human interaction, rather than replace it. This approach can improve teacher-student relationships, reduce anxiety, and increase students’ willingness to communicate ( Guan et al., 2025). However, the integration of AI in managing student welfare in Indonesia remains very limited. Many schools have not yet integrated AI into their administrative systems for student counseling services, and therefore, the technology’s benefits in violence prevention have not been fully realized.

This is where the quality of counseling administration comes into play. Collaborating with the school isn’t just about student data collection, but also about how well the school can process and manage information and data, collaborate with teachers, counselors, and the principal, and practice case management ( Chuang & Liu, 2022; Li-Min & Hsiu-Hao, 2022; Lowery et al., 2020; Solmonson, 2024; Yordy et al., 2022). This component contributes to creating a healthy mental environment and prioritizing psychological affirmations to support students’ academic achievement. The success of school counseling administration when integrated with AI will help detect and monitor trustworthy data to create healthy schools. It also provides early protection from risky behaviors that can lead to unwanted negative behaviors.

Furthermore, the successful implementation of AI technology and the administrative management of school counseling will not be successful without the support of the school organization. Such support can come in the form of policies and commitments from the principal to encourage the use and provision of digital counseling services for students to increase accessibility and effectiveness ( Çela et al., 2025; Madeline, 2025). Schools with collaborative and well-being-focused cultures will seek to integrate AI into counseling and violence prevention services. This aims to enhance services aimed at addressing student mental health crises by providing supportive real-time guidance and early identification of mental health issues ( Al-Shraifin et al., 2025; Aziz et al., 2025; Baksi et al., 2025; Guo & Hou, 2021), thus creating a healthy and safe school environment. Therefore, the combination of AI, school counseling administration, and organizational support forms a solid, interdependent unit.

**Figure 1.**
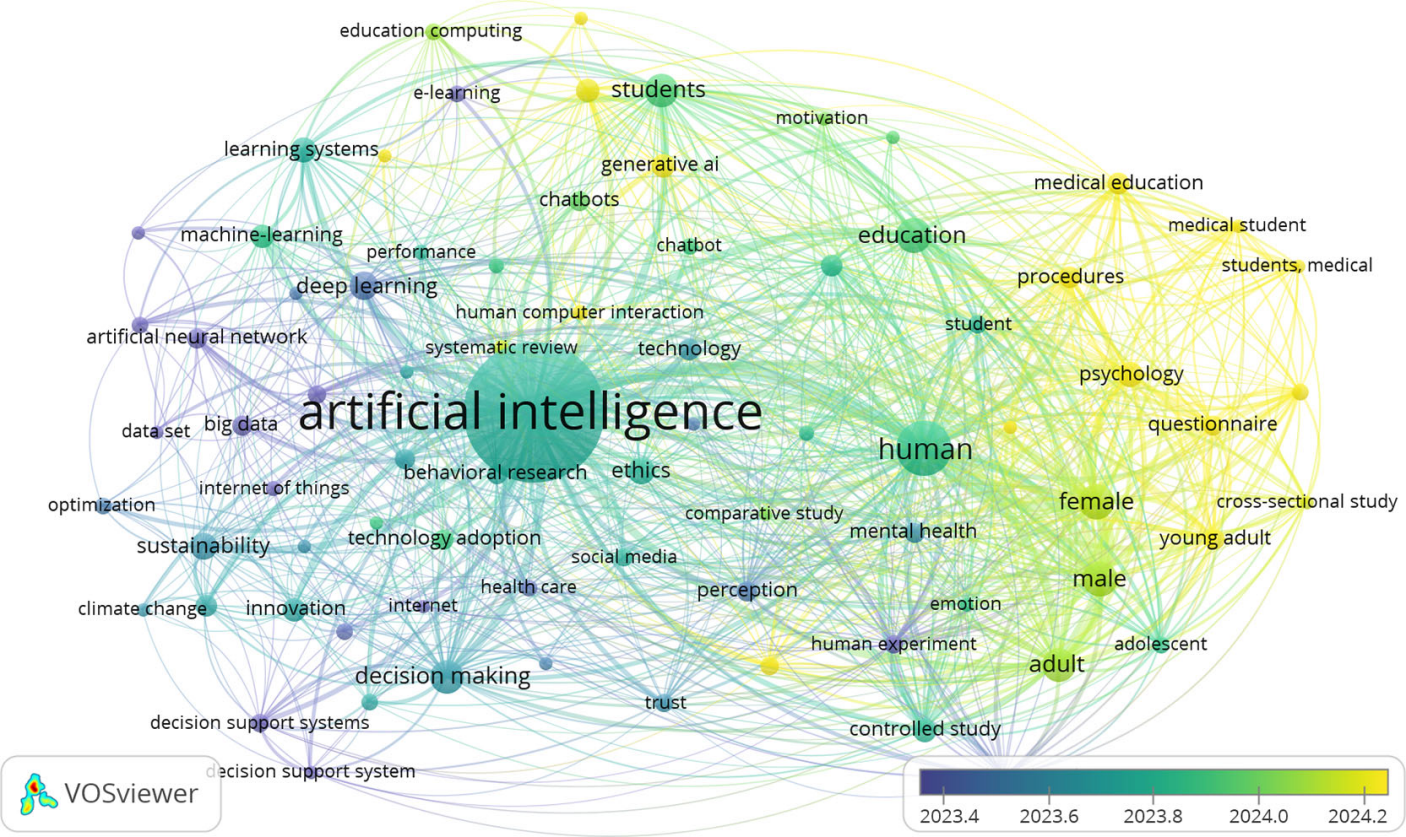
VOSViewer analysis of the last 5 years.

Figure 1 illustrates the bibliometric network visualization generated using VOSviewer for publications over the last five years, research on the use of AI in education has continued to grow. Based on a bibliometric analysis using VOSviewer, data covering 17,660 articles and keywords such as AI, school counseling services, school violence, students, education, bullying, and mental health, the analysis shows that the yellow cluster surrounding the keywords students, education, psychology, and adolescents indicates that in the past two years (2023–2024), there has been increased attention, but research remains limited. However, one interesting aspect of the mapping results is the absence of keywords related to school violence, early risk detection, school counseling, and AI. This indicates that while there is a wealth of research related to AI and education, there is little research on AI for school counseling service management systems. Thus, the VOSviewer results highlight a research gap that remains largely unexplored: how schools use AI as a tool to support counseling services to detect risks and prevent violence in schools.

This study has provided more detailed information related to gaps or branches in bibliometric research as presented in the VOSviewer map above. This study has integrated AI along with the quality of administrative services in school counseling and school organizational support to see how the three interact in improving risk identification and early violence prevention in Indonesian secondary schools. Establishing AI as a variable that is more than just a technological tool but as a component of the counseling service system along with school administrative leadership that supports organizational policies. This is rare in international research. The context of secondary schools in Indonesia has not been widely explored as shown in the VOSviewer analysis, particularly regarding student protection, counseling, and violence prevention. This study is expected to answer how AI can be a valuable resource in preventing violence in schools in real terms and not only on paper or as a conceptual framework. This research can add to the global discourse regarding the use of AI in the administrative practices of school counseling services and student protection policies in secondary schools in Indonesia.

## Method

### Research framework

This study uses a quantitative approach to examine the integration of AI, quality, guidance and counseling administration, and school organizational support in strengthening early risk detection in school violence and violence mitigation processes in Indonesian secondary schools. The study population was junior and senior high school students in Indonesia, using a purposive sampling technique, obtaining 619 active student respondents. Respondents were given a questionnaire via Google Forms online. This method considers ease of access for students, as users of digital devices for educational purposes. The questionnaire consisted of five main constructs that were processed using a five-point Likert scale. Questions were designed in a simple, easy-to-understand format and adapted to students’ cognitive abilities. The collected data were then analyzed using Structural Equation Modeling–Partial Least Squares (SEM-PLS) SMART PLS 4. This analysis was chosen because it can handle research model problems containing complex latent variables, many indicators, and non-normally distributed data. This helps researchers determine the relationship between variables directly and indirectly through mediation.

### Research hypothesis

Hypothesis 1: Artificial Intelligence (X1) has a positive effect on Early Risk Detection (Y). This hypothesis states that there is a positive effect of the use of Artificial Intelligence (AI) in the context of understanding counseling services on early detection of the risk of violence in schools. Digital technology such as the use of school websites, WhatsApp, chatboxes and short message services (SMS) for consultations regarding student counseling guidance can help accelerate the identification of early warning indicators of potential problems such as bullying, violence, dropping out of school, and other psychosocial problems. The use of this technological assistance is an objective signal to improve the quality of prevention of early detection of the risk of violence in schools.

Hypothesis 2: The quality of counseling administration (X2) positively contributes to early risk detection (Y). In this case, the tendency of quality counseling administration services such as case tracking, case follow-up, and good data management contributes to increasing the accuracy of responses and the speed of information at the early risk detection and intervention stage. Previous research shows that planned and structured counseling administration services will significantly increase the accuracy and responsiveness of information and encourage positive student development and address psychopathic behaviors, such as anxiety, depression, and other antisocial personality disorders ( Fahmi et al., 2024; Xiong et al., 2023).

Hypothesis 3: Organizational Support (X3) is positively correlated with Early Risk Detection (Y). This premise analyzes that school organizational support, such as policy considerations on the use of AI for school counseling, provision of counseling resources, and teacher training, positively contributes to the school’s ability to detect early risks of violence in schools ( Eutsler et al., 2025; Mason et al., 2024; Su et al., 2024). With a supportive and responsive school environment, teachers and counselors can more quickly and easily identify, respond to, and take preventive action related to risk indicators ( Efthymiou et al., 2025; Sidhu & Sidhu, 2025; Wouters, 2025).

Hypothesis 4: Mediation relationship between latent variables, namely Quality of Counseling Administration (X2) mediates the influence of AI (X1) on Early Risk Detection (Y), Quality of Counseling Administration (X2) mediates the influence of AI (X1) on Organizational Support (X3), Organizational Support (X3) mediates the influence of AI (X1) on Early Risk Detection (Y), Organizational Support (X3) mediates the influence of Quality of Counseling Administration (X2) on Early Risk Detection (Y), AI (X1) strengthens Quality of Counseling Administration (X2) and Organizational Support (X3) in Early Risk Detection (Y).

Hypothesis 5: X1/X2/X3 has a significant influence on Y. This discussion examines the influence of the use of AI (X1) in administering school counseling (X2), and school support organizations (X3) on Early Risk Detection (Y) of school violence. In other words, if X1/X2/X3 is implemented well, Early Risk Detection (Y) of reducing school violence will be optimal.

### Measurement instrument

**Figure 2.**
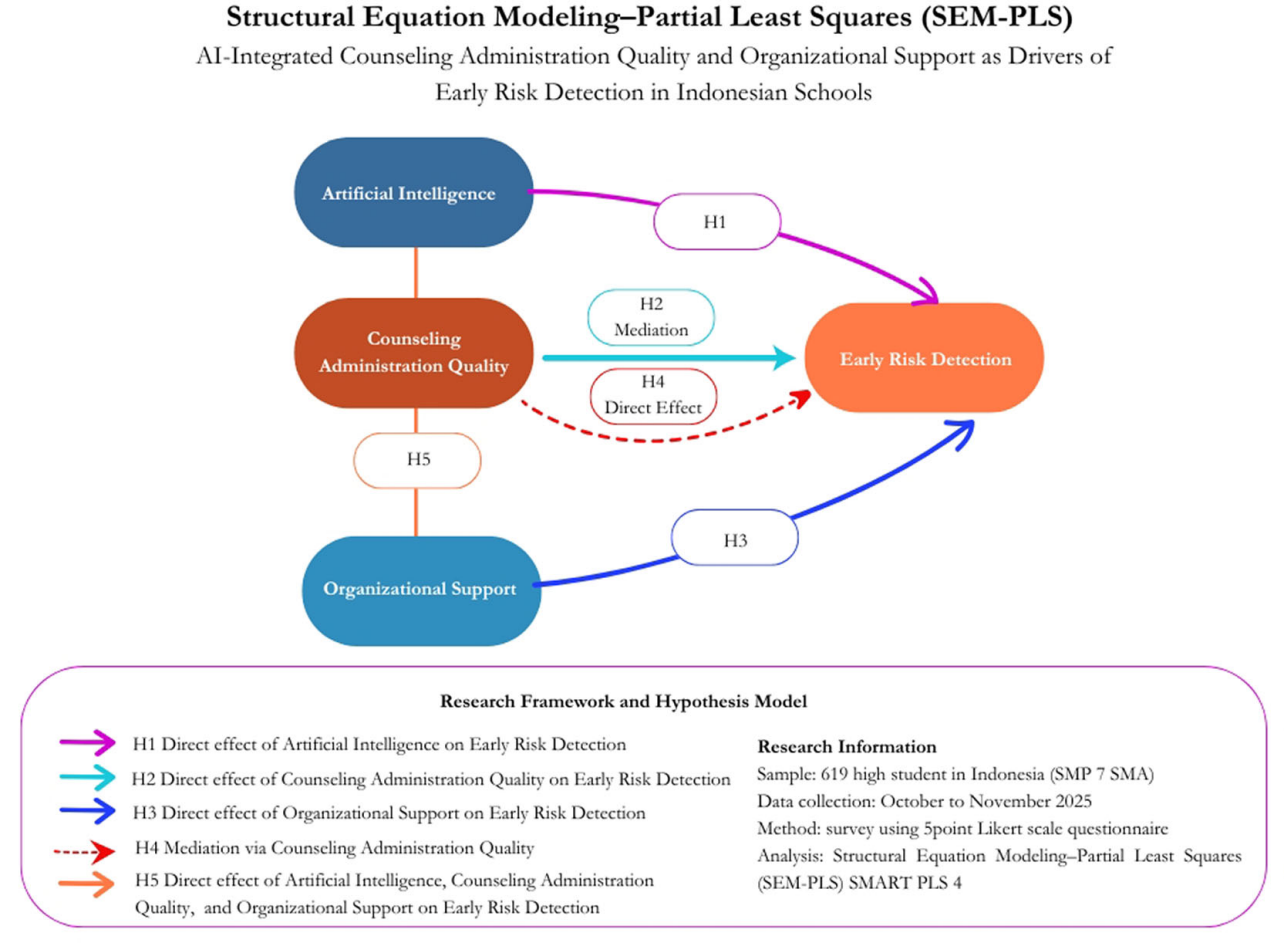
Research framework and hypothesis model.

This study used a Likert scale consisting of five answer choices ranging from 1 to 5. This scale allows researchers to convert subjective responses into measurable data for statistical analysis ( Koo & Yang, 2025; Kusmaryono et al., 2022). In addition, this scale was chosen because it is easy to administer, measure, and code. Data obtained from the Likert scale is ordinal ( Huh & Gim, 2025) which is then processed with Structural Equation Modeling–Partial Least Squares (SEM-PLS) SMART PLS. As illustrated in Figure 2, the proposed research framework and hypothesis model outline the relationships among the variables examined in this study.

## Result and discussion

### Result

**Reflective measurement model assessment**

Following established PLS-SEM guidelines, we assessed indicator reliability, internal consistency reliability, and convergent validity ( Cheung et al., 2024; Hair et al., 2022; Sarstedt et al., 2022). All outer loadings exceeded 0.605 ( Table 1), which is above the acceptable minimum of 0.40–0.70 and indicates that the indicators adequately represent their respective constructs. Composite Reliability (CR) values ranged from 0.760 to 0.870, well above the recommended threshold of 0.70, thus confirming strong internal consistency reliability. The Average Variance Extracted (AVE) values ranged between 0.489 and 0.554 (the construct’s convergent validity is somewhat poor), although slightly below the minimum threshold of 0.50, nevertheless, the p-value was 0.000. Constructs with AVE slightly below 0.50 are still tolerable if the Composite Reliability and indicator loadings are sufficiently high and consistent. ( Amaral et al., 2023; Zulkifli & Zin, 2024). Taken together, these results demonstrate that the measurement model meets the accepted standards for construct validity and reliability in SEM-PLS.

**Table 1.** Measurement model and assessment.

Constructs	Outer loadings	T statistic	VIF	Cronbach’s alpha	Composite reliability	AVE
Artificial intelligence integration				0.760	0.760	0.512
The school provides online services that make it easier for students to find information about help or counseling	0.616	19.490	1.192			
Digital features such as class WA groups help me to consult comfortably	0.700	21.705	1.328			
Technologies such as school portals or online messaging make the process of providing counseling assistance to students faster	0.759	31.982	1.668			
The use of technology for guidance and counseling in schools helps create a sense of comfort for students	0.725	24.132	1.555			
The digital technology provided by the school helps me consult with the guidance counselor more quickly whenever needed	0.768	26.812	1.611			
Counseling administration quality				0.850	0.851	0.489
The guidance counselor at school is easy to contact when I need help	0.673	21.073	1.571			
The guidance teacher explains how to ask for help if there is a problem	0.708	28.341	1.719			
I feel comfortable talking to the guidance counselor about personal issues	0.631	18.888	1.419			
The guidance counselor responds quickly if a student needs support	0.751	34.295	1.990			
Information about the schedule or BK services is easy for students to find	0.670	20.840	1.538			
The guidance counselor provides assistance to students who are experiencing difficulties	0.775	39.928	2.056			
Guidance teachers maintain the confidentiality of students who tell them	0.673	22.743	1.513			
Guidance and counseling services help students understand how to solve problems at school	0.702	24.197	1.598			
Organizational Support				0.837	0.846	0.509
Schools support students to feel safe while learning	0.605	14.510	1.352			
Teachers at school encourage students	0.763	32.755	1.814			
School rules are made to protect students	0.712	21.078	1.650			
The school provides positive extracurricular activities for students	0.639	14.563	1.552			
If students consult about their problems, the teacher immediately helps	0.813	45.290	2.318			
The school is open to receiving reports if problems occur between students	0.735	27.962	1.843			
The school reminded students not to commit violence	0.707	23.739	1.716			
The school provides a character guidance program so that students behave in a manner that respects each other	0.715	25.415	1.591			
Early Risk Detection				0.861	0.870	0.554
The teacher asked me how I was doing if I looked gloomy or not my usual self	0.778	31.014	2.212			
The teacher reminds students if their grades decrease	0.695	26.124	1.507			
Teachers ask questions if they see students being alone or looking uncomfortable	0.819	50.133	2.267			
Teachers help students who are experiencing problems	0.776	43.567	1.704			
The school provides a WA contact number for students if they need guidance assistance	0.636	20.475	1.369			
The guidance counselor or homeroom teacher tries to ask about the students’ news or condition	0.747	31.696	1.760			

Meanwhile, the Fornell Larcker and HTMT tests are used to ensure that each construct in the model is indeed different from each other and does not measure the same thing (Discriminant Validity). The Fornell Larcker method is used to assess discriminant validity by comparing the average variance extracted (AVE) of each construct with the squared correlation between the constructs. If the AVE is greater than the squared correlation, then discriminant validity is established ( Afthanorhan et al., 2021). Table 2 shows that all constructs meet the discriminant validity requirements based on the Fornell–Larcker Criterion, where each √AVE value is greater than the correlation between the other constructs.

**Table 2.** Fornell–Larcker criterion.

	Artificial intelligence	Counseling administration quality	Early risk detection	Organization support
Artificial intelligence	0.716			
Counseling administration quality	0.586	0.699		
Early risk detection	0.514	0.585	0.744	
Organization support	0.517	0.665	0.687	0.714

Meanwhile, the Heterotrait-Monotrait Ratio (HTMT) is used to assess discriminant validity by comparing the correlation ratios between constructs. It is very helpful in SEM and has been proven effective in various conditions ( Roemer et al., 2021; Yusoff et al., 2020). Based on Table 3, the results of the discriminant validity test using HTMT show that all HTMT values are below the threshold of 0.90. Thus, each construct in the model has an adequate level of difference from one another. This indicates that discriminant validity has been met.

**Table 3.** Heterotrait-Monotrait Ratio (HTMT).

	Artificial intelligence	Counseling administration quality	Early risk detection	Organization support
Artificial intelligence				
Counseling administration quality	0.728			
Early risk detection	0.640	0.687		
Organization support	0.630	0.767	0.781	

**Structural model assessment**

The results of the model fit evaluation using PLS-SEM in Table 4 show that the SRMR value for the saturated model and the estimated model is 0.075. This score is still below the threshold of 0.08, indicating that the model has a good fit, meaning that the predicted covariance matrix and the empirical covariance matrix show a good level of alignment. Therefore, it can be concluded that the model does indeed show a good fit. Furthermore, the model fit based on d_ULS and d_G, the values from the original sample d_ULS = 2.101 and d_G = 0.481 are above the 95% and 99% confidence intervals of the bootstrap results, respectively. This indicates that on both of these measures the model is confirmed to be not fully aligned (misfit) because the original value is above the upper limit of the confidence interval.

**Table 4.** Structural model fit.

	Saturated model	Estimated model	95%	99%
	Original sample (O)	Sample mean (M)		
SRMR	0.075	0.075	0.046	0.048
d_ULS	2.101	0.717	0.813	0.859
d_G	0.481	0.188	0.215	0.227

However, referring to Hult et al. (2021), the d_ULS and d_G values are sensitive to model complexity and are not the main criteria for assessing the overall model fit ( Hult et al., 2021). In practice, SRMR is the primary measure used by PLS-SEM analysts, while d_ULS and d_G are only additional measures. Considering these points, it is fair to conclude that the model fit is still good and can be used for further analysis including structural model assessment evaluation, even though d_ULS and d_G indicate model misfit, because the SRMR values are still within the acceptable range.

The results of the structural model test in Table 5 indicate that all hypothesized relationship pathways have a significant influence. Artificial Intelligence (AI) in this case has a strong influence on the quality of counseling administration (β = 0.586; T = 20.883; p < 0.000), which means that the application of AI in the management of counseling administration services is efficient, accurate, and of good quality. In addition, AI has a positive and significant influence on organizational support (β = 0.194; T = 4.041; p < 0.000) and early risk detection (β = 0.163; T = 4.337; p < 0.000). Although the direct influence of AI on early risk detection is statistically significant, its influence is relatively smaller compared to all other pathways. This indicates that the role of AI in risk detection is not independent but depends on the existence of supporting mechanisms within the organizational system.

**Table 5.** Structural direct effect model.

	Path coefficient	Sample mean (M)	Standard deviation (STDEV)	T statistics	P values
	β values				
Artificial intelligence - > counseling administration quality	0.586	0.588	0.028	20,883	0.000
Artificial intelligence - > early risk detection	0.163	0.163	0.038	4,337	0.000
Artificial intelligence - > organization support	0.194	0.192	0.048	4,041	0.000
Counseling administration quality - > early risk detection	0.159	0.160	0.049	3,221	0.001
Counseling administration quality - > organization support	0.552	0.555	0.048	11,519	0.000
Organization support - > early risk detection	0.497	0.498	0.042	11,977	0.000

In contrast, the influence of counseling administration quality on organizational support (β = 0.552; T = 11.519; p < 0.000) was quite significant. This proves that counseling administration quality is a strong foundation in strengthening organizational systems, policies, and coordination. Furthermore, counseling administration quality also has a positive effect on early risk detection (β = 0.159; T = 3.221; p = 0.001), indicating that a quality counseling administration system supports faster and more accurate risk identification. The influence of organizational support on early risk detection is strong and significant (β = 0.497; T = 11.977; p < 0.000). This indicates that the success of early risk detection is largely determined by organizational readiness, policy support, leadership, infrastructure, and coordination between units.

On the other hand, Table 6 illustrates the specific indirect effects of all mediation paths in the structural model, which are statistically significant. Artificial Intelligence has an indirect effect on Early Risk Detection through Counseling Administration Quality and Organizational Support. Furthermore, Counseling Administration Quality also mediates the relationship between Artificial Intelligence and Organizational Support. Sequential mediation was also found through Counseling Administration Quality and Organizational Support in optimizing the role of Early Risk Detection. These findings also support the role of Counseling Administration Quality and Organizational Support as important mediators in improving early detection of the risk of violence in schools in the context of Artificial Intelligence-based counseling administration.

**Table 6.** Structural model of specific indirect effects.

	Path coefficient	Sample mean (M)	Standard deviation (STDEV)	T statistics	P values
	Original sample (O)				
Artificial intelligence - > counseling administration quality - > early risk detection	0.093	0.094	0.030	3.113	0.002
Artificial intelligence - > counseling administration quality - > organization support	0.323	0.326	0.034	9,565	0.000
Artificial intelligence - > organization support - > early risk detection	0.096	0.096	0.026	3,647	0.000
Artificial intelligence - > counseling administration quality - > organization support - > early risk detection	0.161	0.162	0.019	8,278	0.000
Counseling administration quality - > organization support - > early risk detection	0.274	0.276	0.030	9,112	0.000

**Figure 3.**
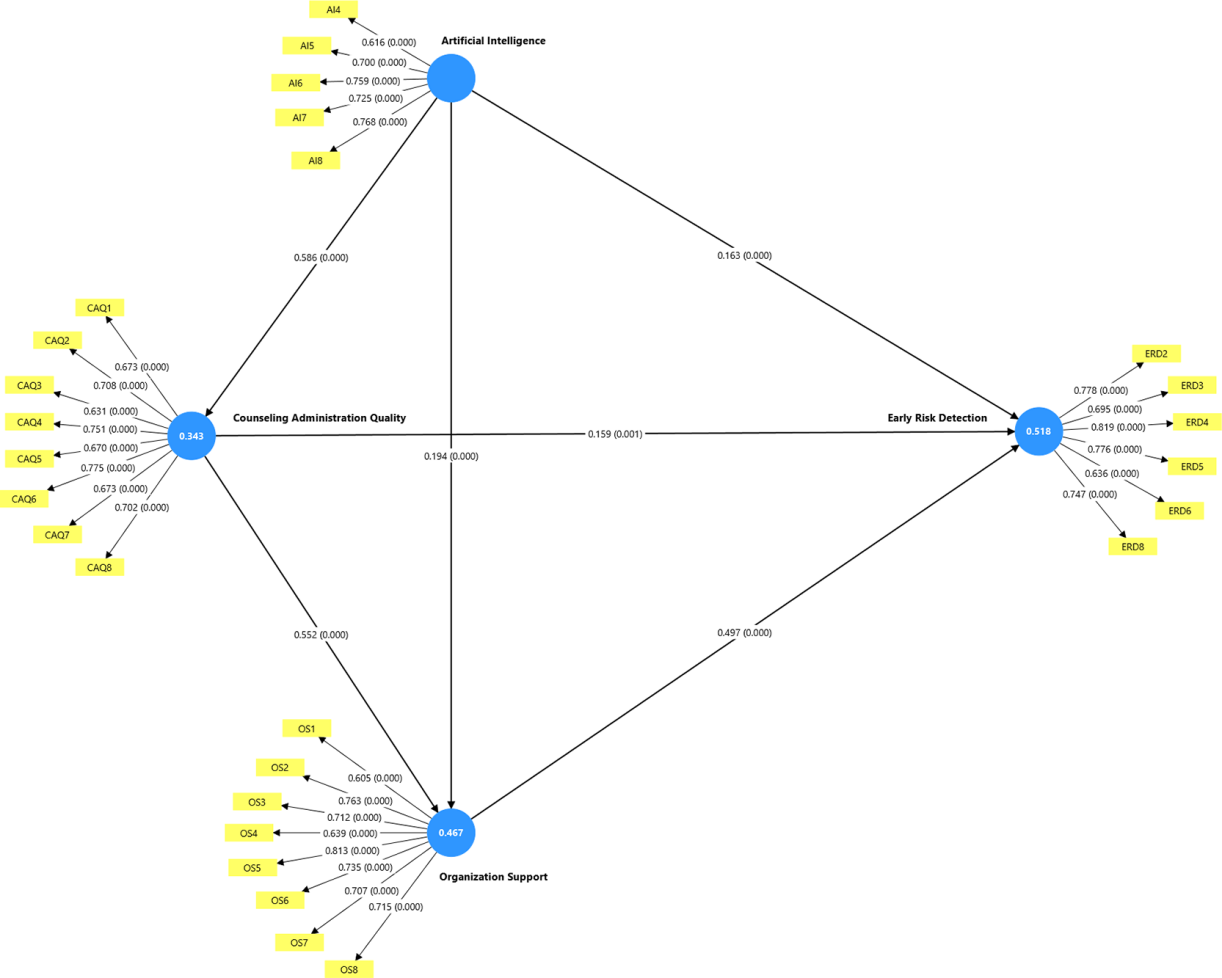
Results of PLS SEM evaluation.

Figure 3 shows the results of the SEM-PLS analysis of all structural paths in the model, which have a positive and statistically significant effect. Artificial Intelligence has the greatest effect on Counseling Administration Quality (β = 0.586; p < 0.001) and a positive effect on Organizational Support (β = 0.194, p < 0.001) and Early Risk Detection (β = 0.163; p < 0.001). Meanwhile, Counseling Administration Quality also has a significant effect on Organizational Support (β = 0.552; p < 0.001) and Early Risk Detection (β = 0.159; p = 0.001), while Organizational Support has a significant effect on Early Risk Detection (β = 0.497; p < 0.001).

The coefficient of determination explains that the model has a negative explanatory power at a moderate to substantial level on Counseling Administration Quality (R ^2^ = 0.343), Organization Support (R ^2^ = 0.467), and Early Risk Detection (R ^2^ = 0.518). On the other hand, the results of specific indirect effects show that Artificial Intelligence has a significant indirect effect on Early Risk Detection through Counseling Administration Quality (β = 0.093; p = 0.002), and through Organization Support (β = 0.096; p < 0.001), as well as on the hierarchical mediation path of Counseling Administration Quality and Organization Support (β = 0.161; p < 0.001) which indicates that there is partial mediation in the model.

## Discussion

The greatest impact of AI on the quality of counseling administration (β = 0.586) indicates that technologies such as AI have significantly improved the efficiency of counseling services. This finding aligns with previous research, where AI plays a role in analyzing and diagnosing data and discovering patterns missed in traditional analysis ( Garcia, 2025; Sun et al., 2025). In addition, AI in the case management system is also useful in helping to schedule consultation sessions and reminders, thereby reducing the significant administrative burden in the process of implementing school counseling services ( Yu Wu et al., 2025). Technology acts as a powerful facilitator in improving the accuracy, efficiency, and integration of counseling service data ( Muhammad, 2024). By leveraging advanced AI-based data analytics, digital tools, and comprehensive data systems, counselors can provide more accurate, efficient, and accessible services.

On the other hand, the direct effect of artificial intelligence on early risk detection was smaller, at β = 0.163. This finding reinforces the view in the educational technology literature that the effectiveness of AI-based systems is highly dependent on the implementation context and organizational readiness. Successful AI implementation requires a robust technology infrastructure and compatibility with existing systems. Challenges include integration with legacy systems, data quality issues, and the complexity of AI systems ( Madanchian et al., 2025; N. M. et al., 2025; Pathak & Bansal, 2025). In this context, the mediating role of the quality of counseling administration and significant organizational support reinforces socio-technical systems theory, which asserts that the recursive interaction between technology and people, within the context of social practices and norms, is crucial for effectively utilizing IT resources. This perspective helps in understanding how organizational and technological resources interact to improve performance ( Kaur et al., 2024). The large coefficient of organizational support’s influence on early risk detection (β = 0.497) indicates that supportive policies, leadership, and organizational culture are instrumental in enabling AI analysis to be transformed into preventive action. This is in line with Sulaymani et al. (2022) who stated that before intelligent technology can be adopted in educational institutions, structural support is needed so that this intelligent technology can contribute to strategic decision-making ( Sulaymani et al., 2022).

The significant mediating role of administrative quality of counseling and organizational support in this model supports the strengthening of the socio-technical systems theory approach, which states that technological performance is a product of the interaction between technical and social components in an organization ( Bali Kamra Professor et al., 2024; Davis et al., 2025). The large coefficient of influence of organizational support on early risk detection (β = 0.497) indicates that organizational support is critical as a form of analysis of the output of the AI analytical model system into a useful preventive measure. This is in line with the results of previous research that the adoption of intelligent technology in educational institutions requires structural support to contribute to strategic decision-making ( George & Wooden, 2023; Rico-Bautista et al., 2020).

The R ^2^ coefficient value of 0.518 means that 51.8% of the total change in early risk detection of school violence can be explained by a combination of variables: artificial intelligence, quality of counseling administration, and organizational support. These results indicate that the model has moderate explanatory power. This further confirms that the approach taken is more integrative than purely deterministic models that rely solely on technology. This also provides a positive contribution to previous research that has only placed AI in a dominant position and has not explicitly recognized the contribution of organizational support in early detection of school violence.

Overall, this research supports the view that the strategic value of applying AI to educational administration lies in its ability to improve administrative efficiency and early risk detection for school violence prevention. AI drives decision-making through proactive risk management, thereby leading to a more resilient education ecosystem. The positive impact of AI in the education system is undeniable, and the need for ethical considerations and practical challenges to address this need to be addressed ( Kilianova et al., 2025; Mitchell, 2025; Pavunraj et al., 2025).

## Conclusion

AI performs well in detecting early risks of violence in secondary schools, particularly when coupled with a counseling service administration system and organizational support from the school. The use of AI has the potential to detect risks earlier, with the greatest potential for AI to function within a more structured administrative system, supported by relevant school policies, leadership, and resources. This finding is considered to almost confirm that the risks detected are not solely due to technology, but rather to organizational systems that need to be integrated and aligned with technology and administrative systems. Furthermore, in the context of this research, empirically demonstrates that an integrative model connecting AI, the quality of counseling service administration, and organizational support can provide a deeper understanding of how schools function, through a reactive approach, towards proactive, data-driven violence prevention and student protection.

These findings provide practical and theoretical contributions, particularly in the context of the school counseling system in Indonesia, and support the need for responsible use of AI in educational administration. Essentially, this research confirms that to continuously detect risks and prevent violence in schools, relying not only on technology but also on the extent to which school organizations manage, understand, and utilize technology to protect and prioritize student well-being. This study strengthens the socio-technical systems theory regarding the use of AI in supporting early identification of violence risks in schools, however, its effectiveness depends on the administrative quality of guidance and counseling services and organizational support in the form of leadership and policies. The key to success is that technology will only have an impact if its management is combined with a good administrative system. In addition, this study adds insight into the field of educational administration by positioning the administrative quality of guidance and counseling services as a strategic administrative mechanism that bridges the impact of the use of technological innovation and protection from violence against students.

Practically, the results of this study strengthen the quality of administrative services and organizational support for improving school counseling services by adopting the use of AI for early detection of the risk of violence against students. Schools need to develop better counseling systems, focus on case management, and facilitate the provision of ethical and sustainable resources. Furthermore, support in the form of teacher and counselor training and the integration of AI into the counseling service administration system to strengthen detection and enhance effective risk prevention needs to be enhanced.

## Ethics statement

The Research Ethics Committee of the Indonesian University of Education granted permission for this study before data collection (Approval Number: 072/ UN.40.EC/KP.09.03/2026). This study was conducted in accordance with the research guidelines of the ethics body and international ethical standards for research involving humans.

## Informed consent

Data collection involved respondents aged 13–18 years. Therefore, written informed consent was obtained from the participants’ parents or legal guardians before they participated in the study. Permission to conduct the study was also granted by the relevant school institution. All participants were given an explanation of the study’s purpose and provided written informed consent before completing the questionnaire. Participants’ responses were collected anonymously and used for research purposes.

## Data Availability

The dataset supporting the findings of this study is available in the Zenodo repository. The data consist of coded questionnaire responses, summary descriptive statistics (including number of observations, mean, standard deviation, minimum, and maximum values), and demographic distribution of respondents by age and gender. This dataset forms the basis for the statistical analyses reported in this study. The dataset can be accessed at: https://doi.org/10.5281/zenodo.18890605 Novebri, N. (2026). Dataset for: AI-Integrated Counseling Administration Quality and Organizational Support as Drivers of Early Risk Detection in Indonesian Schools [Data set]. Zenodo. https://doi.org/10.5281/zenodo.18890605 To maintain respondent confidentiality, the dataset does not contain any personally identifiable information.
