# Supplementary material for: AI-Integrated Counseling Administration Quality and Organizational Support as Drivers of Early Risk Detection in Indonesian Schools
Source: F1000Res. 2026 Jun 5;15:585. Originally published 2026 Apr 19. [Version 2] doi: 10.12688/f1000research.178853.2 (PMC13261190; doi:10.12688/f1000research.178853.2)
Supplement: Peer review history

# Reviewer report: Reviewer response for version 2

**DOI:** 10.5256/f1000research.202643.r491254

**Published:** 2026-06-13

**Authors:** Yiying Xiong

**Affiliations:** 1 A Johns Hopkins School of Education, Maryland, USA

Approve

Is the work clearly and accurately presented and does it cite the current literature?

Partly

If applicable, is the statistical analysis and its interpretation appropriate?

Yes

Are all the source data underlying the results available to ensure full reproducibility?

Yes

Is the study design appropriate and is the work technically sound?

Yes

Are the conclusions drawn adequately supported by the results?

Yes

Are sufficient details of methods and analysis provided to allow replication by others?

Partly

Reviewer Expertise:

My research includes scalable interventions such as AI integration into counseling and counselor education.

I confirm that I have read this submission and believe that I have an appropriate level of expertise to confirm that it is of an acceptable scientific standard.

# Reviewer report: Reviewer response for version 2

**DOI:** 10.5256/f1000research.202643.r492186

**Published:** 2026-06-11

**Authors:** Nuansa Bayu Segara

**Affiliations:** 1 Universitas Negeri Surabaya, Surabaya, East Java, Indonesia

This manuscript presents a very interesting and up-to-date study. The method used to analyze the data is in accordance with the research objectives, and the data were analyzed appropriately and in accordance with the procedures in PLS. However, some of the methods are still unclear, as some explanations are deemed insufficient. First, the data collection in this purposive survey research needs to be questioned. The reason for the purposive method must be explained. What criteria were used to determine the sample of 619? Second, the data validity and reliability procedures must be written. Third, what parameters (requires references and values ​​for each parameter) are used to determine the validity of the test values ​​from each estimate.

Is the work clearly and accurately presented and does it cite the current literature?

Yes

If applicable, is the statistical analysis and its interpretation appropriate?

Yes

Are all the source data underlying the results available to ensure full reproducibility?

Yes

Is the study design appropriate and is the work technically sound?

Yes

Are the conclusions drawn adequately supported by the results?

Yes

Are sufficient details of methods and analysis provided to allow replication by others?

Partly

Reviewer Expertise:

educational research, model of teaching

I confirm that I have read this submission and believe that I have an appropriate level of expertise to confirm that it is of an acceptable scientific standard.

## References

[1] : Modelagem de Equações Estruturais com Utilização do Smartpls. *Revista Brasileira de Marketing* .2014;13(2) : 10.5585/remark.v13i2.2717 56-73 10.5585/remark.v13i2.2717

[2] : PLS-SEM STATISTICAL PROGRAMS: A REVIEW. *Journal of Applied Structural Equation Modeling* .2021;5(1) : 10.47263/JASEM.5(1)06 i-xiv 10.47263/JASEM.5(1)06

# Reviewer report: Reviewer response for version 1

**DOI:** 10.5256/f1000research.197291.r480437

**Published:** 2026-05-25

**Authors:** Yiying Xiong

**Affiliations:** 1 A Johns Hopkins School of Education, Maryland, USA

This is a very timely and interesting topic. However, there may need to be more information in the introduction and methods section. In the introduction section, you may add more research about how AI chatbots are being utilized with mental health needs. You may cite the following articles for this part. In the methods section, the authors will need to add more detailed information about how AI is being integrated into their detection system.

Is the work clearly and accurately presented and does it cite the current literature?

Partly

If applicable, is the statistical analysis and its interpretation appropriate?

Yes

Are all the source data underlying the results available to ensure full reproducibility?

Yes

Is the study design appropriate and is the work technically sound?

Yes

Are the conclusions drawn adequately supported by the results?

Yes

Are sufficient details of methods and analysis provided to allow replication by others?

Partly

Reviewer Expertise:

My research includes scalable interventions such as AI integration into counseling and counselor education.

I confirm that I have read this submission and believe that I have an appropriate level of expertise to confirm that it is of an acceptable scientific standard, however I have significant reservations, as outlined above.

## References

[1] : Chatbots for Children and Adolescents: A Meta-analysis of Chatbots for Mental Health Issues and Psychosocial Well-Being Outcomes. *Adolescent Research Review* .2026; 10.1007/s40894-026-00284-8 10.1007/s40894-026-00284-8

[2] : Generative AI Mental Health Chatbots as Therapeutic Tools: Systematic Review and Meta-Analysis of Their Role in Reducing Mental Health Issues. *Journal of Medical Internet Research* .2025;27: 10.2196/78238 10.2196/78238 PMC1270744041401240

# Response

**Published:** 2026-05-27

**Authors:** Novebri Novebri

**Affiliations:** Educational Administration, Universitas Pendidikan Indonesia, Bandung, West Java, Indonesia

Thank you for the constructive feedback. In this revised version, we have strengthened the Introduction by incorporating recent literature on AI-based mental health chatbots, including the suggested references, to better reflect current developments in AI-supported mental health interventions. We have also expanded the Methods section to clarify how AI is operationally integrated into the school-based early risk detection system. These revisions improve the manuscript’s clarity and methodological transparency.

# Reviewer report: Reviewer response for version 1

**DOI:** 10.5256/f1000research.197291.r482236

**Published:** 2026-05-20

**Authors:** Shahida Sajjad

**Affiliations:** 1 Metropolitan University, Karachi, Pakistan

This study presents a fresh perspective on the integration of AI, administrative counseling quality, and school support in early risk detection in secondary school students of Indonesia. The study makes a meaningful contribution to the field of social sciences. Using a survey, this research addresses a gap in existing literature. The author’s approach demonstrates originality and adds value to ongoing scholarly discussions.

 The paper is well‑structured, with a logical flow from introduction to conclusion. Each section builds upon the previous one, making the argument easy to follow. The writing style is clear, concise, and accessible to both specialists and a broader academic audience.

 The literature review is comprehensive and well‑integrated. References are current, relevant, and demonstrate the author’s engagement with key debates in the field. This situates the study within a strong scholarly context.

 The methodology is sound and appropriate for the research objectives. The author has carefully selected methods that align with the study’s aims, and the data collection and analysis are executed with rigor. This strengthens the reliability of the findings. The statistical tests were suitable for the data type and research hypotheses.

 The results are presented clearly, with sufficient detail to support the conclusions. The discussion is insightful, linking findings to existing literature while highlighting the study’s unique contributions. The author successfully demonstrates the practical and theoretical implications of the research.

 This is a well‑written and thoroughly researched paper and reflects academic integrity, careful analysis, and a commitment to advancing knowledge.

Is the work clearly and accurately presented and does it cite the current literature?

Yes

If applicable, is the statistical analysis and its interpretation appropriate?

Yes

Are all the source data underlying the results available to ensure full reproducibility?

Yes

Is the study design appropriate and is the work technically sound?

Yes

Are the conclusions drawn adequately supported by the results?

Yes

Are sufficient details of methods and analysis provided to allow replication by others?

Yes

Reviewer Expertise:

General Education, Special Education, and leadership.

I confirm that I have read this submission and believe that I have an appropriate level of expertise to confirm that it is of an acceptable scientific standard.

# Response

**Published:** 2026-05-20

**Authors:** Novebri Novebri

**Affiliations:** Educational Administration, Universitas Pendidikan Indonesia, Bandung, West Java, Indonesia

Thank you very much for the positive and encouraging feedback. We greatly appreciate the reviewer’s valuable comments and recognition of our study’s contribution and rigor.
